# The influence of insulin and incretin-based therapies on renal tubular transport

**DOI:** 10.1007/s40620-024-02048-w

**Published:** 2024-08-21

**Authors:** Erica Rosati, Gianfranco Di Giuseppe, Teresa Mezza, Pietro Manuel Ferraro

**Affiliations:** 1 U.O.S. Terapia Conservativa della Malattia Renale Cronica, Dipartimento di Scienze Mediche e Chirurgiche, Fondazione Policlinico Universitario A. Gemelli IRCCS, Rome, Italy; 2 Dipartimento Universitario di Medicina e Chirurgia Traslazionale, Università Cattolica del Sacro Cuore, Rome, Italy; 3 Endocrinologia e Diabetologia, Fondazione Policlinico Universitario Agostino Gemelli Istituto di Ricovero e Cura a Carattere Scientifico (IRCCS), Rome, Italy; 4 Dipartimento di Medicina e Chirurgia Traslazionale, Università Cattolica del Sacro Cuore, Rome, Italy; 5 Digestive Disease Center, Pancreas Unit, Fondazione Policlinico Universitario Agostino Gemelli IRCCS, Rome, Italy; 6 Section of Nephrology, Department of Medicine, Università degli Studi di Verona, Verona, Italy

**Keywords:** Tubular transport, Electrolytes handling, Insulin, Glucagon-like peptide-1, Serine protease dipeptidyl peptidase-4

## Abstract

The tubular function of the kidney is very complex and is finely regulated by many factors. These include a variety of hormonal signaling pathways which are involved in the expression, activation and regulation of renal transporters responsible for the handling of electrolytes. Glucose-lowering drugs such as insulin and incretin-based therapies, exert a well-known renal protective role in diabetic kidney disease, mainly acting at the glomerular level. In the literature, several studies have described the effect of insulin and the incretin hormones on tubular transport. Most of these studies focused on the variations in excretion and clearance of sodium but did not extensively and systematically investigate the possible variations that these hormones may induce in the tubular regulation of all the other electrolytes, urea metabolism, acid–base balance and urinary pH. While insulin action on the kidney is very well-described, the renal tubular impact of incretin-based therapies is less consistent and the results available are scarce. To our knowledge, this is the first review summarizing the effects induced on renal tubules by insulin, glucagon-like peptide-1 (GLP-1) receptor agonists and serine protease dipeptidyl peptidase-4 (DPP4) inhibitors in both healthy and diabetic human subjects. This is significant because it highlights the existence of a renal-gut and pancreas axis which also has a direct tubular effect and enables a deeper understanding of renal physiology.

**Graphical abstract:**

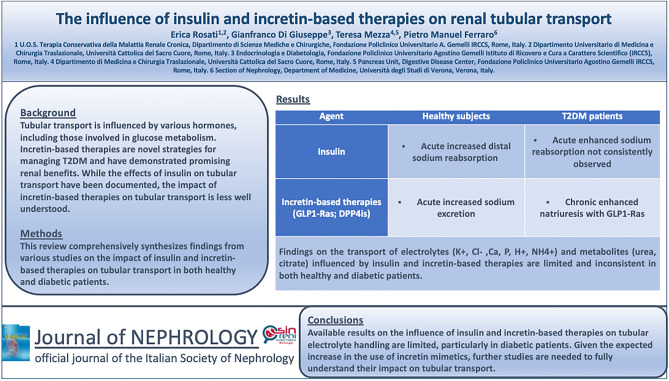

## Introduction

The renal tubular transport system is highly sophisticated, relying on a complex renal architecture and being regulated by systemic hemodynamics, hormones, and nerves for proper function [1].

Various hormonal pathways influence the expression and function of renal transporters, including those that regulate glucose metabolism.

Insulin plays a primary role as a regulator of glycemic status and overall energy homeostasis in the liver, adipose tissue and muscle. Under physiological conditions, this hormone also has secondary effects on the kidneys thanks to the expression of insulin receptor substrates in renal cells, which ensure a role in metabolism, electrolyte and acid–base regulation and absorption of filtered substances [2]. Numerous studies over the past decades have demonstrated the localization of insulin receptor substrates in the proximal tubule, thick ascending limb, distal convoluted tubule and collecting duct [3].

In animal studies, insulin seems to increase sodium reabsorption along all the tubular segments, and the antinatriuretic action of insulin was confirmed in humans [4].

Even if sodium handling is the best described effect, insulin action extends to the fine-tuning of several electrolytes through multiple pathways and transporters.

Type 2 diabetes mellitus (T2DM) is a chronic metabolic disorder characterized by hyperglycemia as a result of the reduced sensitivity of tissues to insulin and beta-cell dysfunction [5–7], eventually leading to insulin secretion deficit [8, 9]. While the influence of insulin on tubular transport in healthy individuals has been investigated, it remains unclear whether the insulin resistance seen in diabetic patients affects only glucose metabolism regulation or if it also impacts tubular transport.

In addition to insulin, the hormones GLP-1 and glucose dependent insulinotropic polypeptide also participate in the regulation of blood glucose levels [10, 11]. These hormones, known as incretins, are secreted from the gut in response to food ingestion and stimulate insulin secretion from pancreatic islets in a glucose-dependent way by binding specific beta-cellular receptors. Once secreted, the incretin hormones are rapidly degraded by serine protease dipeptidyl peptidase-4 [12]. Among incretin-based therapies, GLP-1Ras exert their function via direct binding of ubiquitary GLP-1Rs, while serine protease dipeptidyl peptidase-4-inhibitors increase endogenous incretin hormone half-life by specifically inhibiting the degrading protease [10, 11, 13].

Beyond their effects on the pancreas, incretins can activate specific GLP-1Rs in various organs, including the kidneys. GLP-1Rs are found in the kidney vasculature and numerous human studies have shown their presence both in the glomerulus and in the renal tubule [14, 15].

Growing evidence suggests that GLP-1Ras and serine protease dipeptidyl peptidase-4 inhibitors can affect renal function through both GLP-1R-dependent and independent mechanisms [16]. Although both drug categories regulate glucose homeostasis by activating the incretin receptors, their impact on renal physiology may differ. Indeed, serine protease dipeptidyl peptidase-4 inhibitors degrade various bioactive peptides beyond incretins and this may affect different pathways independently of GLP-1 receptor activation [16].

Incretin-based therapies have become well-established strategies for treating diabetic patients, with GLP-1RAs showing particularly promising beneficial effects on renal outcomes. Consequently, their use is expected to increase significantly. This highlights the need to review the potential impact of these drugs on tubular transport and compare their effects with the better-known impact of insulin in both healthy and diabetic patients.

### Regulation of tubular transport of sodium and water by insulin

All the results concerning insulin impact on tubular transport are shown in Table 1.

**Table 1:** Acute effects of Insulin on tubular transport

Author	*N*	Insulin	Study population	Intervention	Duration	Na^+^	K^+^	PO_4_^3−^	Ca^2+^	H^+^	NH_4_^−^	Citrate	H_2_O
Acute effects													
DeFronzo [4]	6	Insulin 2 mU/Kg/min IV	Healthy subjects	Euglycemic- hyperinsulinemic clamp	1-day trial	↓C ↓AE ↓FE	↓AE	↓C ↓AE	↑C ↑AE				↑CH_2_0 = V = uFR
Skott [18]	8	Sequential dosages of insulin 20–40 mU/m^2^/min IV	Healthy subjects	Euglycemic- hyperinsulinemic	1-day trial	↓C (Ins 40) ↓FE (Ins 40) = PRR = DRR = FPR ↑FDR (Ins 40)	↓C (Ins 40)	= C					= PRR = DRR = FPR = FDR
Gans [19]	9	Sequential dosages of insulin 50–300-500 mU/Kg/h IV	Healthy subjects	Euglycemic- hyperinsulinemic vs placebo	1-day trial	↓FE							
Friedberg [17]	6	Insulin 40 mU/Kg bolus + 1 mU/Kg/min IV	Healthy subjects	Hyperinsulinemic-euglycemic clamp	1-day trial	↓FE	↓FE						
				Hyperinsulinemic-euglycemic clamp + potassium infusion	1-day trial	= FE	= FE						
Ter Maaten [20]	20	Sequential dosages of insulin 50- 150 mU/kg/h IV	Healthy subjects	Euglycemic- hyperinsulinemic clamp	1-day trial	↓FE ↓FPR ↑FDR							= uFR
Ter Maaten [21]	10	Sequential dosages of insulin 50- 150 mU/kg/h IV	Healthy salt-sensitive hypertensive subjects (SS)	Euglycemic- hyperinsulinemic clamp	1-day trial	↓FE = FPR ↑FDR							
	10		Healthy salt-resistant hypertensive subjects (SR)	Euglycemic- hyperinsulinemic clamp	1-day trial	↓FE (ins. 50) = FE (ins. 150) = FPR ↑FDR							
Abate [42]	35	Insulin 80 mU/m^2^/min IV	Healthy subjects	Hyperinsulinemic euglycemic clamp	1-day trial					↑pH	↑U	↑U	
Skott [22]	18	Insulin 40 mU/m^2^/min IV	T2DM patients (10) vs Healthy subjects (8)	Euglycemic- hyperinsulinemic clamp	1-day trial	↓C ↓FE = PRR = DRR = FPR FDR	↓C						= PRR = DRR = FPR = FDR
Pelikánová [23]	11	Insulin 1 mU/Kg/min IV Insulin 0.3–1.1 U/h IV	Insulin-dependent diabetic patients	Hyperinsulinemic euglycemic clamp with Intralipid vs isoinsulinemia + intralipid	1-day trial	= C = FPR ↑FDR ↑ADR							↑V
	24	Insulin 0.3–1.1 U/h IV (only diabetic)	Insulin-dependent diabetic patients (11) vs healthy subjects (13)	Isoinsulinemia + saline	1-day trial	↓C ↑FPR = APR = FDR = ADR							= V

*T2DM* Type 2 diabetes mellitus, *AE* absolute excretion, *FE* fractional excretion, *C* clearance, *FPR* fractional proximal reabsorption, *FDR* fractional distal reabsorption, *APR* absolute proximal reabsorption, *ADR* absolute distal reabsorption, *CH20* free water clearance, *V* urine volume, *uFR* urine flow rate, *PRR* proximal reabsorption rate, *DRR* distal reabsorption rate, *U* urinary concentration, *IV* intravenous

*Acute effects of insulin in healthy subjects* DeFronzo et al. [4] were among the first to study the effect of insulin on tubular function in humans; in this study, 6 healthy subjects underwent a euglycemic-hyperinsulinemic clamp and they showed a significant reduction of absolute and fractional sodium excretion as well as a reduction in sodium clearance during insulin administration as compared to the controls. These findings were accompanied by an increase in free water clearance despite the unchanged urine flow rate and urine volume. This study demonstrates an effect of insulin on urinary sodium excretion which is independent of changes in the filtered load of glucose, glomerular filtration rate (GFR), renal blood flow, plasma aldosterone concentration and volume depletion. The proximal reabsorption of sodium is expected to reduce urinary volume and free water clearance, while in all study subjects, urine volume was unchanged and free water clearance increased [4]. According to the authors, these findings are consistent with an insulin action in the distal nephron (i.e., the ascending limb of the Loop of Henle and/or the distal convoluted tubule) [4].

Other authors demonstrated the reduction of fractional sodium excretion in healthy subjects during a euglycemic-hyperinsulinemic clamp, but this result was completely abolished when potassium was infused during the clamp, preventing insulin-induced hypokalemia, thus suggesting that the antinatriuretic effect of insulin may be indirectly linked to hypokalemia [17].

Several groups investigated the tubular excretion of sodium in subjects undergoing euglycemic-hyperinsulinemic stimulation with sequential dosages of insulin ([18–21]). Skøtt and colleagues demonstrated an insulin-induced sodium retaining-effect acting distally in the tubule in human: by measuring lithium clearance, they were able to show a reduction in sodium clearance and a significant increase of fractional distal reabsorption of sodium in an insulin dose-dependent way, while fractional proximal sodium reabsorption and water reabsorption rate remained unchanged across the whole tubule length [18].

Euglycemic-hyperinsulinemic stimulation in healthy individuals with three sequential dosages of insulin (50, 300 and 500 m-units h ^−1^ kg ^−1^) resulted in markedly reduced fractional sodium excretion in insulin-treated subjects compared to baseline: in particular, the decrease in fractional sodium excretion was greater during infusion of the intermediate than the lowest dose of insulin (*P* = 0.05). In contrast, during infusion of the highest dosage of insulin, the reduction in fractional sodium excretion was similar to the value obtained with the lowest dosage [19].

Ter Maaten and colleagues found that insulin induces renal sodium retention by increasing fractional distal tubular sodium reabsorption, despite a decrease in fractional proximal tubular reabsorption [20], a result not consistent with other studies. To investigate whether hyperinsulinemia contributes to the development of salt-sensitive (SS) hypertension, 10 non-diabetic subjects with salt-sensitive hypertension (SS group) and 10 salt-resistant hypertension subjects (SR group) underwent sequential insulin clamp techniques [21]. In healthy subjects, an increase in GFR and a decrease in proximal tubular reabsorption are expected compensatory mechanisms to counteract insulin-mediated sodium retention. In contrast, in the previous study, salt sensitivity was associated with an impaired decrease in fractional proximal tubular reabsorption during insulin infusion [20].

The exposure to physiological and supraphysiological doses of insulin led to increased fractional distal tubular sodium reabsorption and decreased fractional sodium excretion in both groups (except for supraphysiological dose of insulin which was not associated with changes in fractional sodium excretion in the salt-resistant group). No significant changes in fractional proximal tubular reabsorption were found in either group, arguing against a role for insulin in the development of salt-sensitive hypertension [21].

In conclusion, trials investigating the acute effects of insulin on tubular function in healthy individuals offer valuable insights. Despite the infusion of different dosages of insulin, the majority of studies support the notion of a distal nephron action of insulin, as demonstrated by opposite variations of fractional lithium excretion and fractional sodium excretion and direct measurement of reduced fractional proximal excretion and enhanced distal excretion of sodium. However, findings regarding fluid balance are less consistent. In one case, the antinatriuretic effect of insulin was prevented by concomitant potassium infusion, suggesting that natriuresis may indirectly depend on insulin-induced hypokalemia.

*Acute effects of insulin in diabetic subjects* During hyperinsulinemic clamp in T2DM patients with peripheral insulin resistance, Skøtt et al. found an increased distal reabsorption of sodium coupled with reduced fractional sodium excretion and a greater decline in sodium clearance compared to controls [22].

Similarly, Pelikanova et al. [23] demonstrated enhanced sodium reabsorption in the distal tubule in insulin-dependent diabetic patients, with no significant changes in proximal sodium handling following a hyperinsulinemic euglycemic clamp compared to isoinsulinemia.

Conversely, in the same study, the increased distal sodium-retaining effect of insulin was not evident in insulin-dependent patients when compared to healthy controls. Indeed, diabetic patients without microalbuminuria appear to exhibit a propensity towards sodium retention attributed to increased proximal tubular reabsorption of sodium, unaffected by insulin. According to the author, this phenomenon is likely induced by hyperglycemia-stimulated Na + /glucose co-transport activity in the proximal tubule [23].

Overall, the availability of data on the acute effects induced by insulin in diabetic patients is notably limited. The inconsistency of the results may be attributed to several factors, including small sample sizes, variations in insulin dosages, and differences in the population studied, such as insulin-dependent and independent diabetic patients.

### Regulation of tubular transport of sodium and water by incretins

The effects of incretin-based therapies on tubule function are shown in Table 2.

**Table 2:** Acute and chronic effects of GLP-1RA and DPP4-i on tubular transport

Author	*N*	Study population	GLP1Ra/DPP4i	Intervention	Duration	Na^+^	K^+^	Cl^−^	Ca^2+^	H^+^	Urea	H_2_O
Acute Effects												
Gutzwiller [15]	15	Healthy subjects	Synthetic human GLP-1 IV (0.375 pmol/Kg/min)	GLP-1 vs placebo (+ IV hypertonic saline)	1-day trial	↑AE ↑FE						↑V = TcH_2_O
			Synthetic human GLP-1 IV (1.5 pmol/Kg/min)	GLP-1 infusion vs placebo (+ IV hypertonic saline)	1-day trial	↑AE ↑FE						↑V = TcH_2_O
	16	Insulin resistant subjects	Synthetic human GLP-1 IV (1.5 pmol/Kg/min)	GLP-1 vs placebo (+ IV hypertonic saline)	1-day trial	↑AE ↑FE	= AE	↑AE	↑AE	↓AE		↑V = TcH_2_O
Gutzwiller [24]	8	Healthy subjects	Synthetic human GLP-1 IV (1.5 pmol/Kg/min)	GLP-1 vs placebo (+ IV saline load)	1-day trial	↑AE ↑FE	= AE			↑pH_u_		↑V
	9	Healthy subjects	Synthetic human GLP-1 IV (1.5 pmol/Kg/min)	GLP-1 vs placebo (+ oral salt load)	1-day trial	= AE ↑FE	= AE			↑pH_u_		= V
Skov [25]	12	Healthy subjects	Synthetic human GLP-1 IV (1.25 pmol/Kg/min)	GLP-1 vs placebo	1-day trial	↑C	= C		↑C			= uFR
Asmar [29]	7	Healthy subjects	Synthetic human GLP-1 IV (1.5 pmol/Kg/min)	GLP-1 vs placebo	1-day trial	= AE = C	= AE = C			= AE = C		= V
Asmar [30]	8	Healthy subjects	Synthetic human GLP-1 IV (1.5 pmol/Kg/min)	GLP-1 vs placebo	1-day trial	↑AE	= U			= pH_u_		= uFR = CH_2_0
Muskiet [32]	10	Healthy subjects	Exenatide 10 µg IV	Exenatide vs placebo	1-day trial	↑AE ↑FE	= FE			↑pH_u_	= FE	
				Exenatide + L-NMMA vs L-NMMA		↑AE ↑FE	= FE			↑pH_u_	= FE	
Asmar [31]	6	Healthy subjects	Synthetic human GLP-1 IV (1.5 pmol/Kg/min)	GLP-1 vs exendin 9–39 + GLP-1 vs placebo	1-day trial	↑AE	= U			= pH_u_		= uFR = CH_2_0
Asmar [34]	8	T2DM patients	Synthetic human GLP-1 IV (1.5 pmol/Kg/min)	GLP-1 vs placebo (+ glucose clamp)	1-day trial	= AE	= AE			= AE		
Tonneijck [35]	57	T2DM patients	Exenatide 10 µg IV	Exenatide vs placebo	1-day trial	↑AE ↑FE	= AE ↑FE			↑pH_u_	= AE ↓FE	↓uFR ↓CH_2_0
Skov [36]	11	T2DM patients	Liraglutide 1.2 mg SC	Liraglutide vs placebo	1-day trial	↑U = C = AE	↑U = C = AE					= uFR = V
Chronic effects												
Tonneijck [37]	35	T2DM patients	Lixisenatide 20 µg SC	Lixisenatide vs Insulin glulisine	8-week trial	↑FE = AE				↑pH_u_		= uFR
Lovshin [38]	18	T2DM patients	Liraglutide SC (0.6 mg-1.2 mg-1.8 mg)	Liraglutide vs placebo	3-week trial	↑AE						
Tonneijck [39]	56	T2DM patients	Liraglutide 1.8 mg SC	Liraglutide vs placebo	12-week trial	= AE = FE	= AE = FE			= pH_u_	= AE = FE	= uFR = CH_2_0
			Sitagliptin 100 mg oral	Sitagliptin vs placebo	12-week trial	= AE = FE	= AE = FE			= pH_u_	= AE = FE	= uFR = CH_2_0
Lovshin [40]	36	T2DM patients	Sitagliptin 100 mg oral	Sitagliptin vs placebo (+ euglycemic hyperinsulinemic clamp)	1-day trial	↓FE						
					4-week trial	↑FE						

*T2DM* Type 2 diabetes mellitus, *AE* absolute excretion, *FE* fractional excretion, *TcH2O* free water reabsorption, *C* clearance, *PR* proximal reabsorption, *V* urine volume, *uFR* urine flow rate, *U* urinary concentration, *CH20* free water clearance, *IV* intravenous, *SC* subcutaneous

*Acute effect of incretin mimetics in healthy subjects* Initial studies primarily investigated the acute effects of GLP-1 and incretin mimetics on tubular transport in healthy subjects.

Acute infusion of synthetic human GLP-1 acts directly at a proximal level, inducing an increase in absolute and fractional sodium excretion and a rise in urinary volume in both healthy and obese insulin-resistant men [15]. The simultaneous decrease in renal hydrogen ion excretion supports the GLP-1 direct effect on sodium-hydrogen antiporter 3 in the proximal tubular cells [15].

Conversely, when the GLP-1 infusion is associated with an oral salt load instead of an intravenous saline infusion, fractional sodium excretion is still significantly increased, but absolute excretion only increases slightly and there is no variation in the volume of urine. This could be explained if we consider that an oral salt load causes an extracellular volume expansion of a lesser degree than that provoked by intravenous saline infusion [24].

A study by Skov et al. [25], using an isotopic infusion technique coupled with urinary sampling and lithium administration, also showed an increase in renal Na^+^ clearance despite unchanged urine flow rate.

Under Na^+^ standardized conditions, it is generally accepted that the renal clearance and renal extraction of lithium reflect fractional Na^+^ and thereby fluid reabsorption in proximal tubules [26–28].

Since the renal lithium^l^ clearance increased comparably with Na^+^ clearance, the authors suggested a possible GLP-1-mediated natriuresis mechanism located in the proximal tubule.

Asmar et al. did not observe differences in excretion and clearance of sodium in healthy subjects during GLP-1 infusion compared to placebo [29]. Since natriuresis could be elicited by pressure and volume mechanisms, it has to be noted that in the studies of Gutzwiller et al. [15] and Skov et al. [25] the subjects were given NaCl infusions corresponding to a Na^+^ load far greater than that reached in the Asmar study. This could suggest that GLP-1 may contribute to natriuresis induction through volume-regulating mechanisms.

Subsequently, the same authors conducted a randomized controlled study to confirm GLP-1 volume-induced natriuresis. Healthy subjects received a saline load infusion in order to expand their extracellular fluid volume, followed by GLP-1 infusion. They found increased urinary sodium excretion, whereas natriuresis was not associated with increased flow out of the proximal tubule. Additionally, unchanged lithium clearance, urine flow and free water clearance may suggest that proximal transport is not affected during GLP-1 infusion [30]. These data support that the GLP-1 natriuretic effect does not primarily involve inhibition of proximal sodium-hydrogen antiporter 3 activity but is rather mediated by more distal nephron segments. According to the authors, this interpretation is supported by the GLP-1–induced decrease in circulating angiotensin 2 levels. Indeed, extracellular volume regulation appears to be elicited via a tubular mechanism in the distal nephron, secondary to the suppression of angiotensin 2 and independent of changes in total renal plasma flow [30]. The same results were found using the GLP-1 receptor antagonist Exendin 9–39, which abolished the natriuretic effect of GLP-1 during an extracellular fluid volume expansion and did not influence angiotensin 2 levels [31]. These findings may suggest that GLP-1-induced natriuresis is dependent on GLP-1 receptor activation. Furthermore, the study supports a GLP-1 effect mediated by more distal nephron segments, since, during the GLP-1 infusion alone, lithium clearance, urine flow, hydrogen excretion and free water clearance did not change [31].

When exenatide, an incretino-mimetic, is infused in healthy overweight subjects, it induces a rise in fractional and absolute sodium excretion, and the same result was obtained by coupling exenatide infusion with concomitant infusion of the non-selective nitric oxide synthase inhibitor L-NG-monomethyl-arginine, suggesting that exenatide influences renal hemodynamics possibly through interactions with local neurohormonal and vascular factors, such as nitric oxide [32]. Thereby it is shown that acute exenatide administration may induce nitric oxide- and pressure-independent natriuresis.

In healthy subjects, these findings suggest that both GLP-1 and exenatide likely influence renal sodium handling and fluid balance significantly. While most studies report an increase in renal sodium excretion, this effect does not consistently correlate with a parallel increase in urine volume output. It is plausible that GLP-1 and incretin mimetics may operate through mechanisms in both proximal and distal tubules, yet conclusive evidence regarding their precise site of action remains elusive. Furthermore, studies using validated antibodies could not demonstrate the GLP-1R in the proximal tubule [33].

*Acute effect of incretin mimetics in diabetic subjects* In the literature, there are few studies describing the acute effects of GLP-1 analogs and GLP-1Ras on tubular transport in subjects with type 2 diabetes mellitus. A 3-h infusion of synthetic human GLP-1 did not affect the sodium urinary excretion in T2DM patients without nephropathy. An important limitation to this study involved the concomitant euglycemic glucose clamp administered together with GLP-1 infusion, in fact, the resulting threefold increased plasma insulin levels may have led to increased renal sodium reabsorption counterbalancing a possible GLP-1-induced natriuresis [34].

In overweight T2DM patients with normal renal function, the administration of the short-acting GLP-1Ra exenatide resulted in increased sodium excretion, accompanied by a reduction in urine flow rate and free water clearance. No changes in renal hemodynamics were observed except for a significant rise in afferent renal arteriolar resistance [35].

Similarly, a single dose of subcutaneous liraglutide does not affect renal hemodynamics but decreases the proximal tubular sodium reabsorption based on the fractional lithium excretion Besides, liraglutide induces an increase in sodium clearance despite no differences in sodium excretion, urinary flow rate or volume [36]. The majority of liraglutide does not pass the glomerular barrier [36], yet it appears to exert comparable effects on tubular sodium reabsorption to those observed with GLP-1R agonists that undergo filtration, such as synthetic human GLP-1 and exenatide [15, 25, 32]. This finding possibly explains the GLP-1Ras direct inhibition of sodium-hydrogen antiporter 3 as the primary natriuretic mechanism.

In summary, studies examining the acute effects of incretin-based therapies in diabetic subjects show inconsistency. Factors such as the limited number of trials, small sample sizes, and variations in therapies likely impede the establishment of clear evidence.

*Chronic effects of incretin mimetics in diabetic subjects* The 8-week administration of a short-acting GLP-1Ra lixisenatide elicits a natriuretic and urinary alkalizing response in overweight and inadequately controlled T2DM subjects, indicating a possible inhibition of sodium-hydrogen antiporter 3 activity in the proximal tubule mediated by GLP-1R. However, the authors suggest that lixisenatide’s effects may involve the distal tubule since the increased fractional sodium excretion is not associated with changes in (intra-)renal hemodynamic functions through tubulo-glomerular feedback [37]. Additionally, different studies were unable to detect GLP-1R in the tubular lumen [33].

In a 3-week study, the administration of up-titrated doses of the subcutaneous human GLP-1 analog liraglutide elicited significant increases of natriuresis independent of concomitant changes in blood pressure or circulating levels of natriuretic peptides in overweight or obese subjects with T2DM and hypertension [38].

While the effects of GLP-1 on renal tubules were investigated in a larger number of studies, the impact of serine protease dipeptidyl peptidase-4 inhibitors on tubular functions still needs further investigation.

Tonneijck and colleagues [39] investigated the renal effects of sitagliptin or liraglutide in overweight T2DM non-insulin dependent patients without chronic kidney disease. The primary end-point of the study was to assess GFR changes, which were not observed with either therapy or placebo.

Fractional excretion of electrolytes was calculated by using inulin as the reference substance, and no variations in absolute or fractional excretion of sodium were found except for a transient increase of fractional sodium excretion with sitagliptin at week 2, which was not sustained after 12 weeks of treatment.

GLP-1 inhibition of sodium-hydrogen antiporter 3 may induce an initial period of negative sodium balance and a decrease in extracellular volume, followed by compensatory mechanisms such as neurohumoral- and flow-mediated increases in tubular reabsorption [39].

In contrast, different authors found increased fractional sodium excretion after one month of sitagliptin administration compared to an acute single dose or placebo. In this study diabetic patients underwent a standardized liquid meal during which incretin secretion was assessed; further, sitagliptin was administered, followed by a euglycemic clamp [40]. To our knowledge this was the first study demonstrating that sitagliptin induces natriuresis by blocking distal tubular sodium reabsorption. These findings are supported by the lack of change in fractional lithium excretion, which is used as a marker of proximal sodium uptake. Furthermore, Lovshin et al. found that serine protease dipeptidyl peptidase-4 inhibition increases plasma levels of bioactive stromal cell–derived factor-1a1-67, a chemokine with proven natriuretic action in preclinical studies [40].

In conclusion, chronic administration of GLP-1Ras, such as lixisenatide and liraglutide, in overweight or obese subjects with type 2 diabetes mellitus, induces significant increases in natriuresis, suggesting a potential role in renal sodium handling. Studies examining the chronic effects of sitagliptin on renal tubular transport in T2DM patients have yielded inconsistent results, with transient increases in fractional excretion of sodium observed in some cases.

### Regulation of tubular transport of other electrolytes and citrate by insulin

*Potassium* As discussed before, DeFronzo was among the first to study insulin impact on tubular transport in humans, and with regard to potassium, he found a reduction in urinary excretion and plasma concentration. It is well-known that potassium excretion largely depends on its tubular secretion [41].

Since insulin causes an intracellular shift of potassium mainly in liver and skeletal muscle, it is not clear whether the decrease in urinary potassium excretion may be attributed to enhanced potassium transport into tubular cells together with an inhibitory effect of insulin on potassium secretion or to an increase of intracellular potassium in a different compartment [4]. Similarly, Skøtt and colleagues observed a reduced potassium clearance in two different studies in both healthy and diabetic patients treated with euglycemic-hyperinsulinemic clamp [18, 22]. The authors concluded that it is not possible to assess whether insulin has a direct effect on renal tubular potassium secretion and/or reabsorption or whether the observed changes depend on modifications in plasma potassium concentration [18]. Friedberg et al. found decreased fractional potassium excretion in healthy subjects during acute hyperinsulinemia, but when a simultaneous infusion of potassium was administered, the reduction in fractional potassium excretion was surprisingly abolished as was fractional sodium excretion_._ This finding suggests that the antinatriuresis occurring after insulin infusion is at least partly mediated indirectly by acute hypokalemia [17].

*Calcium and phosphate* In healthy subjects, hyperinsulinemic clamp induces increases in absolute excretion and clearance of calcium, in contrast with an antinatriuretic effect. Possible explanations for the increased calcium excretion include a direct inhibitory effect of insulin on calcium transport [4].

In the same setting, a small but significant reduction in plasma phosphate concentration was observed, and the ensuing reduced filtered phosphate load below the tubular reabsorptive capacity could lead to a reduction in phosphate excretion and clearance [4]. In contrast, different authors found no significant changes in phosphate clearance [18].

*Urinary pH, ammonia and citrate* The work of Abate was the only one to explore the impact of insulin on urinary pH, ammonia and citrate in healthy subjects. They found that acute hyperinsulinemia in healthy volunteers is associated with higher urinary pH, urinary ammonia excretion and urinary citrate excretion [42].

### Regulation of tubular transport of other electrolytes and urea by incretins

Almost all studies focus on renal hemodynamics and variation of natriuresis but few data are available regarding the tubular handling of different urinary electrolytes as potassium, calcium, chloride, urinary pH and urea excretion.

*Potassium* Urinary potassium was measured in the majority of studies and no changes in excretion or clearance were found with GLP-1 analogs, GLP-1Ras, serine protease dipeptidyl peptidase-4 inhibitors, regardless of whether the subjects were healthy or diabetic [15, 24, 25, 29, 32, 34, 36, 38, 39]. Different authors found a suppression of plasma levels of angiotensin 2 I[25, 30, 31, 36] independently of changes in total renal plasma flow [30].

The mechanism by which GLP-1 reduces angiotensin 2 is not clear: potentially, GLP-1 can act at several sites that influence angiotensin 2 levels, (such as suppression of angiotensinogen availability and/or angiotensin converting enzyme 1 (ACE-1) activity, or even by increased activity of ACE-2 and neprilysin with enhanced metabolism and clearance of angiotensin 2) [30].

Interestingly, GLP-1-induced angiotensin 2 suppression does not influence aldosterone concentrations and this could explain unchanged urinary potassium excretion.

In contrast, Tonneijck et al. [35] observed an increase in urinary potassium excretion in overweight T2DM men treated with exenatide, indicating that the ratio of sodium reabsorption to potassium secretion is affected in the cortical collecting tubule, as found in preclinical studies.

*Hydrogen and urinary pH* Results relative to changes in hydrogen excretion and urinary pH are not consistent. In less recent studies it was found that GLP-1 was functioning as a proximal diuretic and urinary alkalizer, since GLP-1Ra administration reduces sodium-hydrogen antiporter 3 activity in the apical membrane of proximal tubular cells [24, 32]. Similarly, Tonneijck et al. [37] found lixisenatide has natriuretic and urinary alkalizing effects, suggesting a sustained GLP-1R-mediated inhibition of sodium-hydrogen antiporter 3 activity in the proximal tubule. However the authors cannot exclude distal tubule involvement because of increased fractional sodium excretion not linked to intra-renal hemodynamic variations. Noteworthy, in a previous study the same author did not find changes in urinary pH with either liraglutide or sitagliptin administration [39].

Conversely, Asmar et al. [30, 31] observed that the natriuretic effect of GLP-1 is not associated with variations in hydrogen ion excretion, suggesting that GLP-1 action does not primarily involve inhibition of sodium-hydrogen antiporter 3 activity but rather an effect mediated by more distal nephron segments.

*Urea* Few studies have investigated the impact of incretin-based therapies on urinary excretion of urea.

Muskiet and colleagues [32] found no variation in urea excretion with exenatide, while in a different study exenatide infusion reduced urinary flow, free water clearance and fractional lithium excretion without affecting osmol clearance. These findings could be compatible with increases in vasopressin levels or vasopressin receptor activation. Notably, human studies have shown GLP-1R expression in the supraoptic nucleus [35].

In a different study, Tonneijck et al.[39] found an initial increase of fractional lithium excretion with sitagliptin, which was not sustained and returned to baseline after 12 weeks of treatment.

*Chloride and calcium* Gutzwiller et al. described parallel increases in natriuresis and enhanced chloride and urinary calcium excretion induced by GLP-1. Chloride proximal reabsorption is linked to active sodium transport; likewise, variations in the proximal reabsorption of sodium will affect chloride reabsorption in a similar manner. Calcium transport is almost passive and follows the gradients established by sodium, chloride, and water reabsorption. The increase in urinary calcium excretion in this setting reinforces the proximal inhibitory effect of GLP-1 on tubular sodium reabsorption [15]. Skov et al. [25] confirmed a significant rise in calcium clearance in healthy subjects treated with infusion.

## Conclusion

In healthy subjects, insulin infusion exerts an antinatriuretic effect on distal tubular segments. However, it is not possible to precisely determine the location of insulin receptors in humans. Furthermore, insulin-induced hypokalemia may mediate the role of insulin’s sodium-retaining effect.

In the literature, there is a scarcity of studies investigating the effect of insulin on tubular transport in diabetic patients. Insulin appears to acutely increase distal sodium reabsorption in patients with T2DM, as evidenced by the reduction in urinary sodium excretion observed in insulin-resistant individuals. This suggests a potential antinatriuretic effect of insulin that is independent of the degree of insulin resistance. Nonetheless, there is a lack of evidence regarding the chronic effects of insulin on tubular transport and whether the antinatriuretic effects may be altered by chronic compensatory mechanisms.

The impact of incretins on natriuresis is less consistent. Acute administration of GLP-1 analogs and receptor agonists appears to induce natriuresis in healthy individuals, possibly mediated solely through volume-regulating mechanisms rather than a direct action of GLP-1 on tubular cells. In diabetic patients, chronic use of GLP-1 receptor agonists seems to have a natriuretic effect, while the effects of serine protease dipeptidyl peptidase-4 inhibitors remain unclear.

The specific site of action of GLP-1 agonists is also unclear. Recent findings suggest that GLP-1Ra and serine protease dipeptidyl peptidase-4 inhibitors induce natriuresis by blocking distal tubular sodium reabsorptive mechanisms rather than inhibiting proximal sodium-hydrogen antiporter 3 transporters.

In conclusion, there are limited studies investigating the influence of insulin and incretin-based therapies on tubular electrolyte handling, and the available results are not exhaustive or complete.

Given the strong evidence of the beneficial and protective effects of glucose-lowering therapies on kidney function, particularly new drugs like GLP-1RAs and serine protease dipeptidyl peptidase-4, it is essential to fully understand their mechanism of action, beyond their effects on the glomerulus and on renal hemodynamics.

Further studies are needed to explore the functioning of renal-gut and renal-pancreas axes and to understand the complex hormonal influence on tubular transport.

## Data Availability

All data generated or analyzed during this study are included in this published article.

## Abbreviations

IRSs: Insulin receptor substrates
GLP-1: Glucagon-like peptide-1
GIP: Glucose dependent insulinotropic polypeptide
DPP-4: Serine protease dipeptidyl peptidase-4
GLP-1Ras: GLP-1 receptor agonists
GLP-1Rs: GLP-1 receptors
DPP-4i: DPP-4 inhibitors
cAMP: Cyclic adenosine monophosphate
RPF: Renal plasma flow
GFR: Glomerular filtration rate
NHE3: Sodium-hydrogen antiporter 3
ANP: Atrial natriuretic peptide
FE_Na_: Fractional sodium excretion
FDR_Na_: Fractional distal tubular sodium reabsorption
FPR_Na_: Fractional proximal tubular reabsorption
T2DM: Type 2 diabetes mellitus
Li^+^: Lithium
ANG II: Angiotensin 2
NO: Nitric oxide
FE_Li_: Fractional lithium excretion
FE_K_: Fractional potassium excretion
ACE-1: Angiotensin converting enzyme 1
ACE-2: Angiotensin converting enzyme 2
FE_U_: Fractional excretion of urea

## References

[1] Vallon V (2009) Micropuncturing the nephron. Pflugers Arch 458(1):189–201. 10.1007/s00424-008-0581-7 18752000 10.1007/s00424-008-0581-7 PMC2954491

[2] Pina AF et al (2020) Insulin: Trigger and Target of Renal Functions. Front Cell Dev Biol 8:519. 10.3389/fcell.2020.00519 32850773 10.3389/fcell.2020.00519 PMC7403206

[3] Tiwari S, Riazi S, Ecelbarger CA (2007) Insulin’s impact on renal sodium transport and blood pressure in health, obesity, and diabetes. Am J Physiol Renal Physiol 293(4):F974-984. 10.1152/ajprenal.00149.2007 17686957 10.1152/ajprenal.00149.2007

[4] DeFronzo RA, Cooke CR, Andres R, Faloona GR, Davis PJ (1975) The effect of insulin on renal handling of sodium, potassium, calcium, and phosphate in man. J Clin Invest 55(4):845–855. 10.1172/JCI107996 1120786 10.1172/JCI107996 PMC301822

[5] di Giuseppe G et al (2021) Prediabetes: how pathophysiology drives potential intervention on a subclinical disease with feared clinical consequences. Minerva Endocrinol (Torino) 46(3):272–292. 10.23736/S2724-6507.21.03405-9 34218657 10.23736/S2724-6507.21.03405-9

[6] Mezza T, Cinti F, Cefalo CMA, Pontecorvi A, Kulkarni RN, Giaccari A (2019) β-cell fate in human insulin resistance and type 2 diabetes: a perspective on islet plasticity. Diabetes 68(6):1121–1129. 10.2337/db18-0856 31109941 10.2337/db18-0856 PMC6905483

[7] Mezza T et al (2014) Insulin resistance alters islet morphology in nondiabetic humans. Diabetes 63(3):994–1007. 10.2337/db13-1013 24215793 10.2337/db13-1013 PMC3931397

[8] Mezza T et al (2018) Increased β-cell workload modulates proinsulin-to-insulin ratio in humans. Diabetes 67(11):2389–2396. 10.2337/db18-0279 30131390 10.2337/db18-0279

[9] Brusco N et al (2023) Intra-islet insulin synthesis defects are associated with endoplasmic reticulum stress and loss of beta cell identity in human diabetes. Diabetologia 66(2):354–366. 10.1007/s00125-022-05814-2 36280617 10.1007/s00125-022-05814-2 PMC9807540

[10] Nauck MA, Quast DR, Wefers J, Pfeiffer AFH (2021) The evolving story of incretins (GIP and GLP-1) in metabolic and cardiovascular disease: A pathophysiological update. Diabetes Obes Metab 23(Suppl 3):5–29. 10.1111/dom.14496 34310013 10.1111/dom.14496

[11] Holst JJ (2019) The incretin system in healthy humans: The role of GIP and GLP-1. Metabolism 96:46–55. 10.1016/j.metabol.2019.04.014 31029770 10.1016/j.metabol.2019.04.014

[12] Makino Y, Fujita Y, Haneda M (2015) Dipeptidyl peptidase-4 inhibitors in progressive kidney disease. Curr Opin Nephrol Hypertens 24(1):67–73. 10.1097/MNH.0000000000000080 25415611 10.1097/MNH.0000000000000080

[13] Di Giuseppe G et al (2023) First-phase insulin secretion: can its evaluation direct therapeutic approaches? Trends Endocrinol Metab 34(4):216–230. 10.1016/j.tem.2023.02.001 36858875 10.1016/j.tem.2023.02.001

[14] Crajoinas RO et al (2011) Mechanisms mediating the diuretic and natriuretic actions of the incretin hormone glucagon-like peptide-1. Am J Physiol Renal Physiol 301(2):F355-363. 10.1152/ajprenal.00729.2010 21593184 10.1152/ajprenal.00729.2010

[15] Gutzwiller J-P et al (2004) Glucagon-like peptide 1 induces natriuresis in healthy subjects and in insulin-resistant obese men. J Clin Endocrinol Metab 89(6):3055–3061. 10.1210/jc.2003-031403 15181098 10.1210/jc.2003-031403

[16] Tsimihodimos V, Elisaf M (2018) Effects of incretin-based therapies on renal function. Eur J Pharmacol 818:103–109. 10.1016/j.ejphar.2017.10.049 29066413 10.1016/j.ejphar.2017.10.049

[17] Friedberg CE, Koomans HA, Bijlsma JA, Rabelink TJ, Dorhout Mees EJ (1991) Sodium retention by insulin may depend on decreased plasma potassium. Metabolism 40(2):201–204. 10.1016/0026-0495(91)90175-v 1988777 10.1016/0026-0495(91)90175-v

[18] Skøtt P et al (1989) Effects of insulin on kidney function and sodium excretion in healthy subjects. Diabetologia. 10.1007/BF00274259 2676669 10.1007/BF00274259

[19] Gans RO, Toom L, Bilo HJ, Nauta JJ, Heine RJ, Donker AJ (1991) Renal and cardiovascular effects of exogenous insulin in healthy volunteers. Clin Sci (London, England: 1979). 10.1042/cs0800219 10.1042/cs0800219 1850681

[20] ter Maaten JC, Bakker SJ, Serné EH, ter Wee PM, Donker AJ, Gans RO (1999) Insulin’s acute effects on glomerular filtration rate correlate with insulin sensitivity whereas insulin’s acute effects on proximal tubular sodium reabsorption correlation with salt sensitivity in normal subjects. Nephrol Dial Transplant 14(10):2357–2363. 10.1093/ndt/14.10.2357 10528658 10.1093/ndt/14.10.2357

[21] ter Maaten JC, Bakker SJ, Serné EH, Donker AJ, Gans RO (2001) Renal sodium handling and haemodynamics are equally affected by hyperinsulinaemia in salt-sensitive and salt-resistant hypertensives. J Hypertens 19(9):1633–1641. 10.1097/00004872-200109000-00016 11564984 10.1097/00004872-200109000-00016

[22] Skøtt P et al (1991) Effect of insulin on renal sodium handling in hyperinsulinaemic type 2 (non-insulin-dependent) diabetic patients with peripheral insulin resistance. Diabetologia 34(4):275–281. 10.1007/BF00405088 2065862 10.1007/BF00405088

[23] Pelikánová T, Smrcková I, Krízová J, Stríbrná J, Lánská V (1996) Effects of insulin and lipid emulsion on renal haemodynamics and renal sodium handling in IDDM patients. Diabetologia 39(9):1074–1082. 10.1007/BF00400657 8877292 10.1007/BF00400657

[24] Gutzwiller J-P et al (2006) Glucagon-like peptide-1 is involved in sodium and water homeostasis in humans. Digestion 73(2–3):142–150. 10.1159/000094334 16809911 10.1159/000094334

[25] Skov J et al (2013) Glucagon-like peptide-1 (GLP-1): effect on kidney hemodynamics and renin-angiotensin-aldosterone system in healthy men. J Clin Endocrinol Metab 98(4):E664-671. 10.1210/jc.2012-3855 23463656 10.1210/jc.2012-3855

[26] Pechere-Bertschi A, Sunaric-Megevand G, Haefliger I, Panarello F, Maillard M, Burnier M (2007) Renal sodium handling in patients with normal pressure glaucoma. Clin Sci (Lond) 112(6):337–344. 10.1042/CS20060082 17014421 10.1042/CS20060082

[27] Seidlerová J et al (2006) Association between arterial properties and renal sodium handling in a general population. Hypertension 48(4):609–615. 10.1161/01.HYP.0000240516.60040.ba 16966578 10.1161/01.HYP.0000240516.60040.ba

[28] Shirley DG, Walter SJ, Noormohamed FH (2002) Natriuretic effect of caffeine: assessment of segmental sodium reabsorption in humans. Clin Sci (Lond) 103(5):461–466. 10.1042/cs1030461 12401118 10.1042/cs1030461

[29] Asmar A et al (2015) Renal extraction and acute effects of glucagon-like peptide-1 on central and renal hemodynamics in healthy men. Am J Physiol Endocrinol Metab 308(8):E641-649. 10.1152/ajpendo.00429.2014 25670826 10.1152/ajpendo.00429.2014

[30] Asmar A et al (2019) Extracellular fluid volume expansion uncovers a natriuretic action of GLP-1: a functional GLP-1-renal axis in man. J Clin Endocrinol Metab 104(7):2509–2519. 10.1210/jc.2019-00004 30835273 10.1210/jc.2019-00004

[31] Asmar A et al (2021) The renal extraction and the natriuretic action of GLP-1 in humans depend on interaction with the GLP-1 receptor. J Clin Endocrinol Metab 106(1):e11–e19. 10.1210/clinem/dgaa643 32927478 10.1210/clinem/dgaa643

[32] Muskiet MHA et al (2016) Acute renal haemodynamic effects of glucagon-like peptide-1 receptor agonist exenatide in healthy overweight men. Diabetes Obes Metab 18(2):178–185. 10.1111/dom.12601 26636423 10.1111/dom.12601

[33] Pyke C et al (2014) GLP-1 receptor localization in monkey and human tissue: novel distribution revealed with extensively validated monoclonal antibody. Endocrinology 155(4):1280–1290. 10.1210/en.2013-1934 24467746 10.1210/en.2013-1934

[34] Asmar A et al (2016) Glucagon-like peptide-1 does not have acute effects on central or renal hemodynamics in patients with type 2 diabetes without nephropathy. Am J Physiol Endocrinol Metab 310(9):E744-753. 10.1152/ajpendo.00518.2015 26956188 10.1152/ajpendo.00518.2015

[35] Tonneijck L et al (2016) Acute renal effects of the GLP-1 receptor agonist exenatide in overweight type 2 diabetes patients: a randomised, double-blind, placebo-controlled trial. Diabetologia 59(7):1412–1421. 10.1007/s00125-016-3938-z 27038451 10.1007/s00125-016-3938-z PMC4901099

[36] Skov J et al (2016) Short-term effects of liraglutide on kidney function and vasoactive hormones in type 2 diabetes: a randomized clinical trial. Diabetes Obes Metab 18(6):581–589. 10.1111/dom.12651 26910107 10.1111/dom.12651

[37] Tonneijck L et al (2017) Postprandial renal haemodynamic effect of lixisenatide vs once-daily insulin-glulisine in patients with type 2 diabetes on insulin-glargine: an 8-week, randomised, open-label trial. Diabetes Obes Metab 19(12):1669–1680. 10.1111/dom.12985 28449402 10.1111/dom.12985

[38] Lovshin JA, Barnie A, DeAlmeida A, Logan A, Zinman B, Drucker DJ (2015) Liraglutide promotes natriuresis but does not increase circulating levels of atrial natriuretic peptide in hypertensive subjects with type 2 diabetes. Diabetes Care 38(1):132–139. 10.2337/dc14-1958 25414155 10.2337/dc14-1958

[39] Tonneijck L et al (2016) Renal effects of DPP-4 inhibitor sitagliptin or GLP-1 receptor agonist liraglutide in overweight patients with type 2 diabetes: a 12-week, randomized, double-blind, placebo-controlled trial. Diabetes Care 39(11):2042–2050. 10.2337/dc16-1371 27585605 10.2337/dc16-1371

[40] Lovshin JA et al (2017) Dipeptidyl peptidase 4 inhibition stimulates distal tubular natriuresis and increases in circulating SDF-1α1-67 in patients with type 2 diabetes. Diabetes Care 40(8):1073–1081. 10.2337/dc17-0061 28550195 10.2337/dc17-0061

[41] Palmer BF et al (2021) Clinical management of hyperkalemia. Mayo Clin Proc 96(3):744–762. 10.1016/j.mayocp.2020.06.014 33160639 10.1016/j.mayocp.2020.06.014

[42] Abate N, Chandalia M, Cabo-Chan AV, Moe OW, Sakhaee K (2004) The metabolic syndrome and uric acid nephrolithiasis: novel features of renal manifestation of insulin resistance. Kidney Int 65(2):386–392. 10.1111/j.1523-1755.2004.00386.x 14717908 10.1111/j.1523-1755.2004.00386.x

